# A Population-based Study of Peripartum Cardiomyopathy in Southern Israel: Are Bedouin Women a New High-risk Group?

**DOI:** 10.5041/RMMJ.10331

**Published:** 2018-04-19

**Authors:** Louise Kezerle, Iftach Sagy, Leah Shalev, Offer Erez, Leonid Barski

**Affiliations:** 1 Department of Internal Medicine “F,” Soroka University Medical Center, Beersheva, Israel; 2 Clinical Research Center, Soroka University Medical Center, Beersheva, Israel; 3 Department of Obstetrics “D,” Soroka University Medical Center, Beersheva, Israel

**Keywords:** Epidemiology, peripartum cardiomyopathy, prognostic factors

## Abstract

**Objectives:**

Peripartum cardiomyopathy (PPCM) is a serious complication of pregnancy. Studies investigating the risk factors that worsen outcomes have yielded conflicting results. The goals of this study were to describe the clinical and echocardiographic characteristics of PPCM in a single tertiary center and to determine the prognostic factors associated with persistence of left ventricular (LV) dysfunction in these women.

**Study Design:**

This retrospective cross-sectional population-based cohort study included all patients with PPCM confirmed by echocardiography who delivered at our center from 2004 to 2014. Two groups were compared to determine long-term maternal outcome: (1) those who recovered normal LV function; and (2) those with residual systolic LV dysfunction.

**Results:**

There were 148,994 deliveries during the study period. Of these, 89,196 patients were Bedouin and 59,798 were non-Bedouin. Forty-six patients met the PPCM study inclusion criteria. The PPCM prevalence for the total deliveries was 1:3,239. The PPCM prevalence among Bedouin patients was 1:2,787 versus non-Bedouin patients of 1:4,983 (*P*=0.037). None of the women had pre-existing chronic hypertension, and there was no maternal death. Patients who had severe or moderate LV dysfunction at the clinical presentation of PPCM were less likely to regain normal LV function than those with mild dysfunction (81.2% versus 56.7%, *P*=0.009). Based on initial echocardiogram, a trend toward residual LV dysfunction was noted in patients with a dilated left ventricle as compared to those with a non-dilated left ventricle (18.8% versus 6.7%, *P*=0.32). A hypokinetic right ventricle was found in 15.2% of the women who suffered from PPCM.

**Conclusion:**

In our cohort, Bedouin women may be at increased risk for PPCM, and patients with severe LV dysfunction have a lower chance of recovery from PPCM.

## INTRODUCTION

Pregnancy and childbirth pose a great challenge to the maternal cardiovascular system. The maternal circulation changes physiologically during pregnancy but gradually returns to its non-pregnancy state during puerperium. Peripartum cardiomyopathy (PPCM), however, refers to the development of clinical heart failure with reduced left ventricular ejection fraction (LVEF) in the final stages of pregnancy or in the early period after delivery.^1^ It affects previously healthy women, and its etiology is thus far unknown. The available experimental evidence suggests that multiple risk factors contribute to a common final pathway of myocardial damage caused by enhanced oxidative stress, altered prolactin processing,^2^,^3^ and impaired vascular endothelial growth factor signaling.^4^ Several factors have been proposed to contribute to the development of PPCM: older maternal age,^5^ African descent,^6^,^7^ hypertensive diseases (chronic hypertension, preeclampsia, and eclampsia),^8^ multi-fetal pregnancy,^5^,^9^ long-term oral tocolytic therapy,^10^ and maternal cocaine abuse.^11^–^13^

The reported incidence of PPCM varies largely among countries, ranging from 1:300 live births in Africa and Haiti to 1:4,000 live births in the United States.^1^,^14^,^15^ The outcome of PPCM is also highly variable. Some women present a quick clinical improvement and show significant echocardiographic recovery of left ventricular (LV) function, whereas in others the disease progresses to cardiogenic shock, arrhythmias, and even death.^5^,^16^–^18^ Mortality rates of women with PPCM vary from 4% to up to 19% in the United States and as high as 28% in other regions.^7^,^13^,^18^–^21^ Prognostic factors consistently associated with a higher risk of death are New York Heart Association (NYHA) functional class at presentation,^22^ LVEF ≤25%–30%,^18^,^23^ black race,^16^,^17^,^24^ indigent status,^25^ multiparity,^24^ and age greater than 30.^26^,^27^

Conversely, recovery of LV function occurs more frequently in PPCM than with other types of dilated cardiomyopathies,^28^ with 20% to 60% of affected women showing complete recovery of LV function in some series.^16^,^18^ Lower LVEF, larger LV end-diastolic diameter at diagnosis, and black race appear to be adverse predictors for recovery,^29^–^32^ although there is still uncertainty as to the significance of these findings as parameters that can successfully predict better outcomes in different populations.

The goals of this study were to describe in a population-based cohort the epidemiological, clinical, and obstetrical characteristics of PPCM in southern Israel and to determine the prognostic factors associated with persistence of LV dysfunction in these women.

## METHODS

We conducted a cross-sectional population-based retrospective cohort study. Patients were identified in our electronic database from January 2004 to December 2014 according to the diagnosis of PPCM (ICD-9 674.5) or other cardiovascular diseases complicating pregnancy, childbirth, or puerperium (ICD-9 678.6). The charts of identified patients were reviewed by two independent doctors to determine which ones fulfilled the diagnostic criteria for PPCM according to the 2010 European Society of Cardiology (ESC) Working Group on peripartum cardiology^14^: (1) development of heart failure (HF) toward the end of pregnancy or in the months following delivery; (2) absence of another identifiable cause for the heart failure; and (3) LV systolic dysfunction with a LVEF less than 45%, with the left ventricle being dilated or not dilated. Women with any previous valvular or congenital heart diseases and/or the presence of other severe conditions that justified the reduced cardiac ejection fraction (EF) were excluded. The obstetrical and medical records of patients included in the study were retrieved, and a unified data set was established. As this was a retrospective study, all data were gained from electronic databases only. After patients were identified by their name and official identity card number, each case was randomized and assigned a different study identification number to ensure patient confidentiality. Furthermore, in order to identify possible predictors of poorer recovery, the study cohort was then divided into two groups: (1) those who recovered normal LV function; and (2) those with residual LV dysfunction, moderate or severe. Our institution uses a standardized grading system for LV dysfunction, in accordance with international values: an EF ≤35% is diagnosed as severe LV dysfunction; an EF of 35%–45% is diagnosed as moderate LV dysfunction; and EF of 45%–55% is considered mild LV dysfunction. Since the numerical estimate of EF was not available for every patient record, the independent cardiologists followed the same qualitative grading system according to these definitions.^33^

This study was approved by the ethics committee of the Soroka University Medical Center.

### Statistical Analysis

Theoretic statistics (prevalence and standard deviations) was used for data analysis to describe the demographic, obstetric, and medical characteristics of the population in question. We performed independent *t* tests and chi-square tests to compare continuous and categorical variables between groups of patients with and without residual LV dysfunction on follow-up echocardiogram (echo). Multivariate analysis was not performed due to the small sample size and the difficulty with achieving statistical significance (α less than 0.1). All the statistical analyses were performed using SPSS 20.0 (IBM, Armonk, NY, USA).

## RESULTS

**Figure 1:**
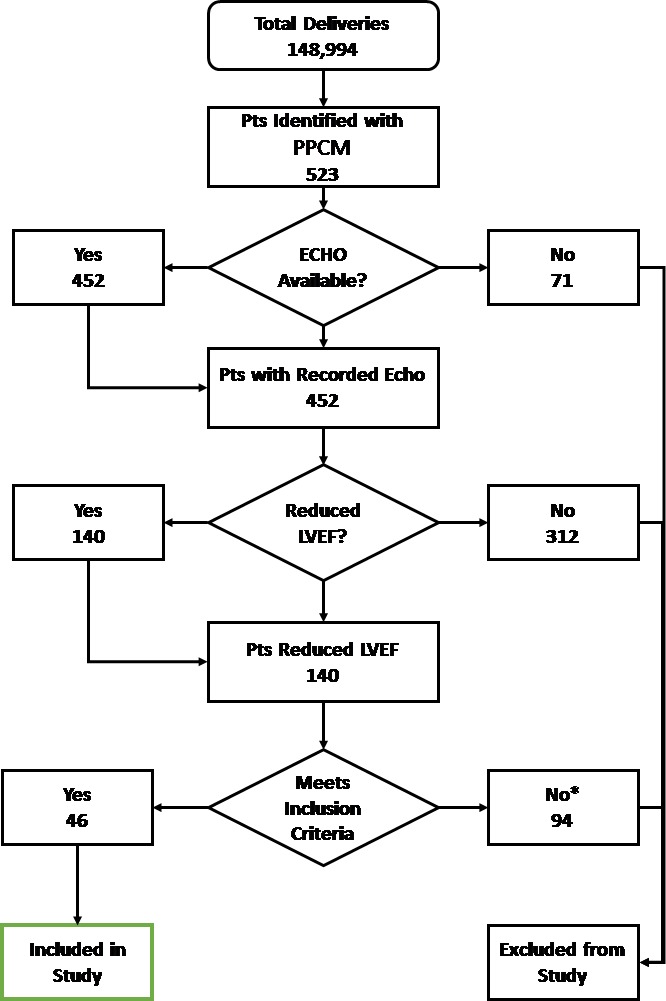
Study Participants Flowchart *74 pts with valvular disease; 9 pts with previous cardiomyopathy; 11 pts with other medical conditions. ECHO, echocardiogram; LVEF, left ventricular ejection fraction; PPCM, peripartum cardiomyopathy; pts, patients.

There were 148,994 deliveries between January 2004 and December 2014 (Bedouin, *n*=89,196; non-Bedouin, *n*=59,798). Based on ICD-9 diagnosis, 523 patients were identified. The full time period for follow-up was not available. After careful review of echo results, only 46 patients (Bedouin, *n*=32; non-Bedouin, *n*=14) met all the eligibility criteria and were included in the statistical analysis. Figure 1 provides a flowchart showing the process for selecting the eligible patients.

The overall PPCM prevalence was 1:3,239 births. Of the 46 patients in the study group, 32 were Bedouin and 14 were non-Bedouin. The prevalence of PPCM among Bedouin women was 1:2,787, whereas the PPCM prevalence among non-Bedouin women was 1:4,983 (*P*=0.037). Table 1 presents the baseline maternal and gestational characteristics for the Bedouin and non-Bedouin women. Bedouin patients showed a trend for younger maternal age, had a lower gestational week, and lower birth weight—although these differences did not reach statistical significance. There were no deaths reported among PPCM patients for the period in question.

**Table 1:** Characteristics of Women with Peripartum Cardiomyopathy (PPCM).

Characteristic	Bedouin (*n*=32)	Non-Bedouin (*n*=14)	*P* Value
Maternal age, mean (SD)	30.4 (6.9)	31.2 (6.7)	0.690
Gestational week, median (IQR)	36.3 (3.6)	37.0 (3.2)	0.542
Birth weight, g (SD)	2,567 (0.893)	2,978 (0.816)	0.148
Gestational number, median (IQR)	5.0 (1.0–8.0)	2.5 (1.0–6.2)	0.244
No. of live birth, median (IQR)	5.0 (1.0–7.0)	2.5 (1.0–5.2)	0.343
APGAR 1 min, median (IQR)	9.0 (5.0–9.0)	9.0 (8.7–9.0)	0.400
APGAR 5 min, median (IQR)	10.0 (9.2–10.0)	10.0 (10.0–10.0)	0.179
Diabetes mellitus, *n* (%)	0 (0.0)	1 (7.1)	0.304
Hypertension, *n* (%)	0 (0.0)	0 (0.0)	1.000
Obesity, *n* (%)	4 (12.5)	2 (14.3)	0.869
Dyslipidemia, *n* (%)	0 (0.0)	1 (7.1)	0.304

IQR, interquartile range.

When comparing the women who had residual LV dysfunction on follow-up echocardiography to those who recovered normal LV systolic function, there were no significant differences in relation to the demographic or obstetric characteristics. Moreover, when comparing other factors previously described as associated with a worse outcome, such as maternal age over 35 and multiparity, no statistical difference was noted between the two groups.

Based on the echo results, the majority of patients had at least moderate LV dysfunction (65.2%), while 15.2% also showed signs of right ventricular (RV) compromise. In comparison to the first echo results, 76% of the patients showed improvement in their LV systolic function on a follow-up echo examination, with 65.2% regaining normal LV function (Table 2).

**Table 2:** Echocardiographic Characteristics of Patients with PPCM.

Characteristic	First Echocardiogram (*n*=46)	Second Echocardiogram (*n*=46)
LV systolic function		
Normal, *n* (%)	None	30 (65.2)
Mild dysfunction, *n* (%)	16 (34.8)	9 (19.6)
Moderate dysfunction, *n* (%)	19 (41.3)	3 (6.5)
Severe dysfunction, *n* (%)	11 (23.9)	4 (8.7)
Regional hypokinesis, *n* (%)*	7 (15.2)	0 (0.0)
Dilated LV, *n* (%)	5 (10.9)	2 (4.3)
Hypokinetic RV, *n* (%)	7 (15.2)	3 (6.5)
Pulmonary HTN, *n* (%)	7 (15.2)	2 (4.3)

* Reference is global hypokinesis.

HTN, hypertension; LV, left ventricle; RV, right ventricle.

When comparing patients with residual PPCM to those without residual PPCM, those with severe and moderate LV dysfunction (13 [81.2%] versus 17 [56.7%], respectively) were more likely to have developed residual damage in the second echo exam (*P*=0.009). There was an overall trend toward residual LV dysfunction in the group of patients with a dilated left ventricle in the initial echo (18.8% versus 6.7%, *P*=0.3) as compared to a non-dilated left ventricle (Table 3).

**Table 3:** Characteristics of the First Echocardiogram in Patients With and Without Residual Peripartum Cardiomyopathy (PPCM).

Characteristics	Residual PPCM (*n*=16)	No Residual PPCM (*n*=30)	*P* Value
Mean time between echo exams, days (SD)	906 (1,001)	694 (1,066)	0.515
LV systolic function			
Moderate or severe LV dysfunction, *n* (%)	13 (81.2)	17 (56.7)	0.09
Moderate dysfunction, *n* (%)	5 (31.2)	14 (46.7)	NA
Severe dysfunction, *n* (%)	8 (50.0)	3 (10.0)	NA
Regional hypokinesis, *n* (%)*	2 (12.5)	5 (16.7)	1.000
Dilated LV, *n* (%)	3 (18.8)	2 (6.7)	0.325
Hypokinetic RV, *n* (%)	2 (12.5)	5 (16.7)	1.000
Pulmonary HTN, *n* (%)	1 (6.2)	6 (20.0)	0.394

* Reference is global hypokinesis.

NA, not applicable.

## DISCUSSION

The prevalence of PPCM in our institution in southern Israel was comparable to the prevalence reported in some centers in the United States, and the disease was less common than in other countries such as South Africa (1:1,000 births) or Haiti (1:300).^1^,^14^,^15^,^34^ The reasons for the difference in prevalence is probably related to ethnic as well as socio-economic factors. As mentioned, African descent is a consistent risk factor for the development of PPCM.^6^,^7^ In our cohort, there was a higher prevalence of the disease among Bedouin women as evidenced by the fact that this ethnic group made up almost 70% of patients diagnosed with PPCM, while constituting only about 40% of the overall maternal population according to local registries over the past few years. Moreover, the prevalence of PPCM was almost two-fold higher in Bedouin women than in the non-Bedouin population. Again, there may be a genetic base for this difference, and local customs may also influence these rates. In a country such as Israel, where there is universal access to health care, socio-economic factors seem to play a smaller role.

The lack of mortality cases evidenced in our study compared to global literature was a surprising finding. We tried to account for this effect by selecting a more general diagnosis and actively reviewing echo results, to find cases that met our inclusion criteria. Another reason for the null mortality rate in our study might be the fact that we excluded patients presenting with another medical diagnosis that could explain the diminished LV systolic function such as severe sepsis or electrolyte imbalances; it is possible that these patients did suffer from severe PPCM that put them at increased risk for an adverse critical illness. Finally, as discussed before, the universal access to medical care might play a role in early diagnosis of the condition, thereby yielding better treatment strategies and closer medical follow-up for PPCM patients.

Some interesting findings arose from the comparison of patients who regained normal systolic LV function to those with residual systolic dysfunction on follow-up echo exams. The small number of cases prevented us from performing additional comparison between the two groups based on the severity of cardiac dysfunction. Of interest, women who had severe disease at presentation also had a lower chance of regaining normal LV function in comparison to those with a milder disease; the same was true for patients with dilated cardiomyopathy as compared to those with a non-dilated left ventricle. When adjusted to maternal age, the degree of LV dysfunction in the first echo remained an independent risk factor for long-term persistence of LV dysfunction. These findings add to the bulk of evidence that point to an association between the severity of LV systolic dysfunction and LV diameter at the time of PPCM diagnosis and the recovery of LV function.^29^–^32^,^34^ Nevertheless, it is important to note that baseline EF has limited sensitivity for prediction of improvement in individual patients, and it should not be used in selecting patients for more aggressive initial therapies.^30^

While much attention has been paid to LV characteristics, only recent studies have examined the importance of RV dysfunction as a potential prognostic factor in PPCM.^35^ The incidence of RV hypokinesis in our study population was 15.2%, while researchers in large prospective cohorts in Nigeria have reported rates of RV dysfunction ranging from 38% to 54% of women suffering from PPCM as well as an association between altered RV function and persistent LV dysfunction.^36^,^37^ Our lower percentage of RV injury is probably due to the fact that the main objective of these studies was to assess RV function specifically, leading to a more focused examination of the right ventricle than would be the case in a routine echo examination. As a result of our small sample size, we did not find an association between the presence of RV hypokinesis and persistence of LV dysfunction on follow-up, but this is a point that should be examined further.

Our study has some strong points and a few disadvantages. First of all, we performed a systematic search of all the patients who had received a diagnosis of cardiovascular disease during pregnancy for the past 11 years in our institution, and actively reviewed all echo results to be sure to include only patients who fit the PPCM criteria and to minimize the effect of selection bias. Secondly, our institution has an electronic database for obstetric and medical data that facilitated a more systematic search for the needed information. Even so, the number of cases was still too small to identify a statistical difference in most variables analyzed. Third, since our research was a retrospective cohort using an electronic database we had no access to detailed clinical complaints, signs, and symptoms. Patients were identified after PPCM diagnosis had already been added to our electronic database. Another disadvantage is that we did not search for outcomes in subsequent pregnancies. In addition, the management protocol for this research was left to the discretion of the treating physician due to the retrospective nature of the electronic database analysis. It should be emphasized that all patients underwent a follow-up echo that allowed for comparison between those who did and did not recover LV function. Finally, the complication rates (other than residual LV dysfunction) were not available from the database. It would have been thought-provoking to describe the rates for arrhythmias and thrombo-embolic events in our PPCM patients, but we were content with mortality rate, since death is the clinical endpoint of interest in these events.

## CONCLUSION

Peripartum cardiomyopathy is a known although rare complication of late pregnancy and early puerperium, with outcomes varying from complete recovery of LV function to severe refractory congestive heart failure leading to heart transplant or even death. Our study has identified that the Bedouin population may be at increased risk for this condition and that a more severe degree of LV dysfunction is probably associated with a reduced chance for full recovery. Moreover, there is a growing need for investigation of the relationship between RV characteristics and PPCM.

## Abbreviations

echo: echocardiogram
EF: ejection fraction
ESC: European Society of Cardiology
HF: heart failure
HTN: hypertension
LV: left ventricular
LVEF: left ventricular ejection fraction
NYHA: New York Heart Association
PPCM: peripartum cardiomyopathy
RV: right ventricular.
